# Crystal structure of *meso*-tetra­kis­(4-nitro­phen­yl)porphyrin nitro­benzene disolvate

**DOI:** 10.1107/S1600536814021503

**Published:** 2014-10-04

**Authors:** Maksym Seredyuk, Elzbieta Gumienna–Kontecka, Anna Brzuszkiewicz, Turganbay S. Iskenderov, Valentina A. Kalibabchuk

**Affiliations:** a National Taras Shevchenko University, Department of Chemistry, Volodymyrska str. 64, 01601 Kyiv, Ukraine; b Faculty of Chemistry, University of Wroclaw, 14, F. Joliot–Curie Str., 50383, Wroclaw, Poland; c O.O. Bohomolets National Medical University, Department of General Chemistry, Shevchenko blvd 13, 01004 Kiev, Ukraine

**Keywords:** crystal structure, porphyrins, hydrogen bonding, supra­molecular chains

## Abstract

The porphyrin core of the title centrosymmetric compound, C_44_H_26_N_8_O_8_·2C_6_H_5_NO_2_, is approximately planar, the maximum deviation being 0.069 (3) Å. The planes of the benzene rings of the nitro­phenyl substituents are almost perpendicular to the porphyrin mean plane, making dihedral angles of 73.89 (9) and 89.24 (9)°. The two pyrrole ring H atoms are equally disordered over the four pyrrole ring N atoms. In the crystal, weak C—H⋯O and C—H⋯N hydrogen bonds link the porphyrin mol­ecules into a three-dimensional supra­molecular network. The nitro­benzene solvent mol­ecules are linked by weak C—H⋯O hydrogen bonds into supra­molecular chains propagating along the *a-*axis direction.

## Related literature   

Porphyrins and metalloporphyrins are of inter­est as building blocks for mol­ecular cages (Meng *et al.*, 2011 ▶), catalysts (Odo *et al.*, 2009 ▶) and photofunctional materials (Yan *et al.*, 2009 ▶). For related structures, see: Silvers & Tulinsky (1967 ▶). For related polymeric complexes, see: Seredyuk *et al.* (2007 ▶); Moroz *et al.* (2012 ▶); Zha *et al.* (2013 ▶). For the synthesis of *meso*-tetra­kis­(4-nitro­phen­yl)porphyrin, see: Bettelheim *et al.* (1987 ▶).
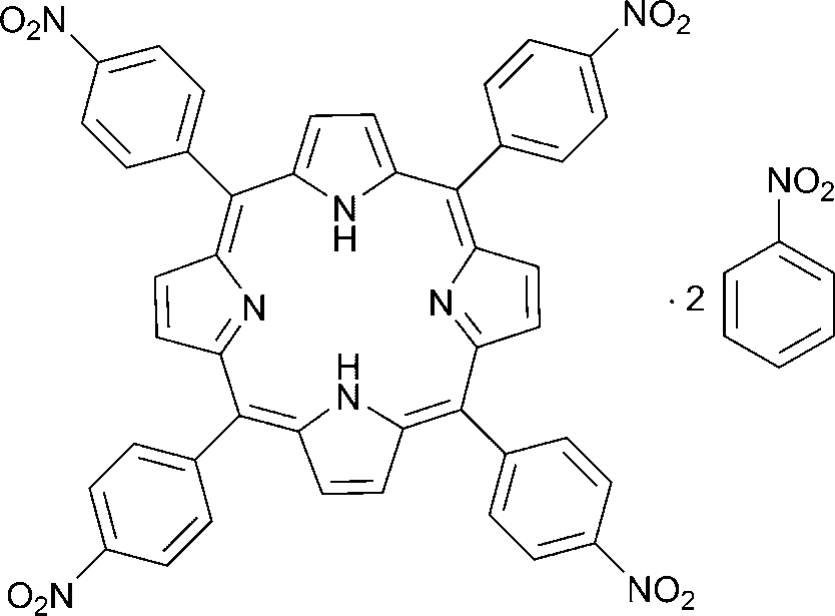


## Experimental   

### Crystal data  

C_44_H_26_N_8_O_8_·2C_6_H_5_NO_2_
*M* *_r_* = 1040.95
Triclinic, 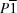

*a* = 7.949 (4) Å
*b* = 10.134 (5) Å
*c* = 16.444 (8) Å
α = 105.43 (5)°
β = 95.37 (4)°
γ = 102.17 (4)°
*V* = 1232.4 (11) Å^3^
*Z* = 1
Mo *K*α radiation
μ = 0.10 mm^−1^
*T* = 293 K
0.13 × 0.09 × 0.03 mm

### Data collection  

Agilent Xcalibur κ-axis diffractometer with a Ruby CCD detector
Absorption correction: multi-scan (*CrysAlis PRO*; Agilent, 2011 ▶) *T*_min_ = 0.981, *T*_max_ = 1.000
10625 measured reflections
5284 independent reflections
3437 reflections with *I* > 2σ(*I*)
*R*_int_ = 0.018

### Refinement  

*R*[*F* ^2^ > 2σ(*F* ^2^)] = 0.086
*wR*(*F* ^2^) = 0.275
*S* = 1.10
5284 reflections
322 parameters
9 restraints
H-atom parameters constrained
Δρ_max_ = 0.60 e Å^−3^
Δρ_min_ = −0.49 e Å^−3^

### 

Data collection: *CrysAlis PRO* (Agilent, 2011 ▶); cell refinement: *CrysAlis PRO*; data reduction: *CrysAlis PRO*; program(s) used to solve structure: *SHELXS97* (Sheldrick, 2008 ▶); program(s) used to refine structure: *SHELXL97* (Sheldrick, 2008 ▶); molecular graphics: *SHELXTL* (Sheldrick, 2008 ▶); software used to prepare material for publication: *SHELXL97*.

## Supplementary Material

Crystal structure: contains datablock(s) I, global. DOI: 10.1107/S1600536814021503/xu5821sup1.cif

Structure factors: contains datablock(s) I. DOI: 10.1107/S1600536814021503/xu5821Isup2.hkl

Supporting information file. DOI: 10.1107/S1600536814021503/xu5821Isup3.cdx

**Fig. 1.**
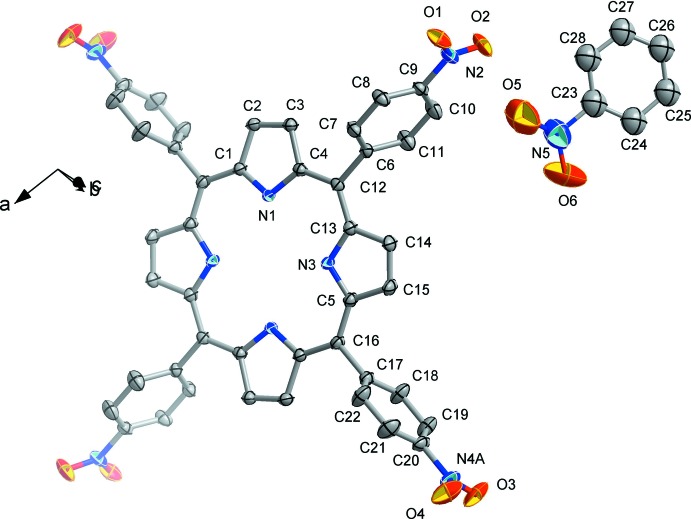
Molecular structure of the title compound with the atom-labeling scheme and 25% probability displacement ellipsoids. Hydrogen atoms are omitted for clarity.

**Fig. 2.**
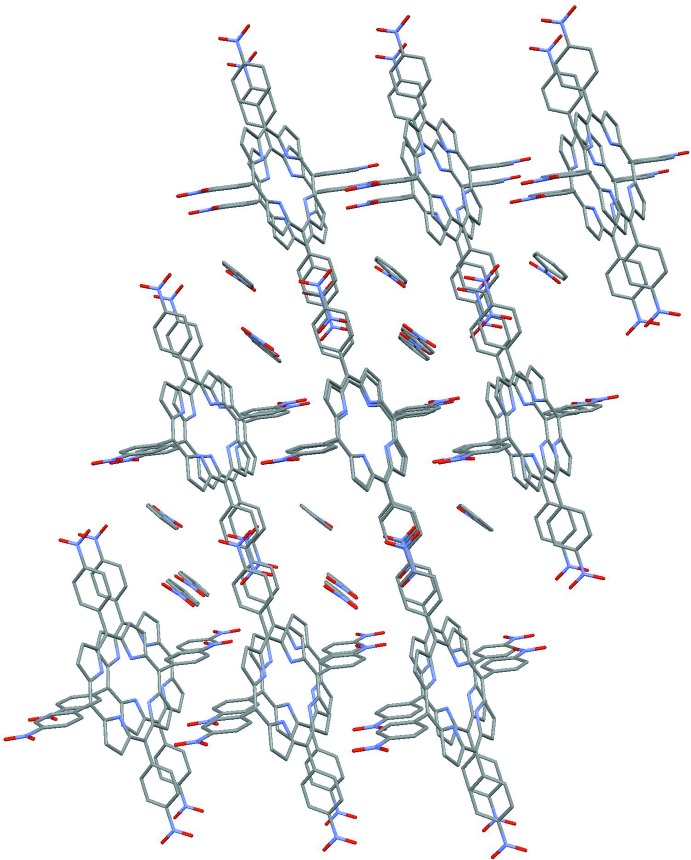
Projection of the crystal packing along a axis.

. DOI: 10.1107/S1600536814021503/xu5821fig1.tif
Mol­ecular structure of the title compound with the atom-labeling scheme and 25% probability displacement ellipsoids. Hydrogen atoms are omitted for clarity.

a . DOI: 10.1107/S1600536814021503/xu5821fig2.tif
Projection of the crystal packing along *a* axis.

CCDC reference: 1026803

Additional supporting information:  crystallographic information; 3D view; checkCIF report

## Figures and Tables

**Table 1:** Hydrogen-bond geometry (, )

*D*H*A*	*D*H	H*A*	*D* *A*	*D*H*A*
C3H3O1^i^	0.93	2.60	3.519(4)	171
C11H11N1^ii^	0.93	2.57	3.426(4)	152
C15H15O3^iii^	0.93	2.56	3.453(5)	161
C18H18O1^iv^	0.93	2.58	3.455(5)	157
C26H26O5^v^	0.93	2.56	3.432(10)	156

Symmetry codes: (i) 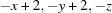; (ii) 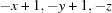; (iii) 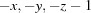; (iv) 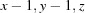; (v) 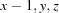.

## supplementary crystallographic information

### S2.1. Synthesis and crystallization

The *meso*-tetra­kis[4-nitro­phenyl]-porphyrin, synthesized according to Bettelheim *et al.* (1987), was crystallized from boiling nitro­benzene as lustrous violet crystals.

### S2.2. Refinement

H atoms were placed in calculated positions with C—H = 0.93 Å and N—H = 0.86 Å, and refined in riding mode with U_iso_(H) = 1.2 U_eq_(N,C). Each amino H atom is equally disordered over two positions.

### S3. Results and discussion

Porphyrins and metalloporphyrins attract attention of the researchers in many aspects, such as building blocks for molecular cages (Meng *et al.*, 2011), catalysts (Odo *et al.*, 2009) or photofunctional materials (Yan *et al.*, 2009). Having continuing inter­est in study of polynuclear complexes (see, for example, Seredyuk *et al.*, 2007, Moroz *et al.* 2012), in this paper we report the structure of *meso*-tetra­kis(4-nitro­phenyl)­porphyrin, a precursor for polytopic bridging ligands (for example, Zha *et al.*, 2013).

The 24-membered porphyrin moiety of the title compound is planar with a maximum deviation of C2 atom equal to ±0.130 (3) Å. The angle between adjacent pyrrole ring planes (C1–C4/N1 and C5–C13/N3) is 7.190 (11), and bond lengths and angles are close to those found for tetra­phenyl­porphyrin (Silvers & Tulinsky, 1967). This suggests that the nitro­phenyl substituents and/or packing effects influence the geometry of the porphyne ring.

The molecule of porphyrin contains two structurally different pairs of pyrrole rings. Despite two hydrogen atoms are disordered over four pyrrole rings, the two structurally non-equivalent pyrrole rings have somewhat different angles C—N—C equal to 107.20 (20)° and 108.30 (20)°.

The 4-nitro­phenyl groups are rotated at angles of 73.89 (9)° and 89.24 (9)° with respect to the porphyrin mean plane, due to steric hindrance with the pyrrole-H atoms of the macrocycle.

The unit-cell packing along the *a* axis is shown in Fig. 2. There are no significant π-π inter­actions between the porphyrins. The 4-nitro­benzene groups around the porphyrin core apparently hinder inter­actions between the porphyrins. The channels formed due to loose packing of porphyrins are occupied by nitro­benzene molecules.

### Crystal data

**Table:**

C_44_H_26_N_8_O_8_·2C_6_H_5_NO_2_	*Z* = 1
*M**_r_* = 1040.95	*F*(000) = 538
Triclinic, *P*1	*D*_x_ = 1.403 Mg m^−^^3^
Hall symbol: -P 1	Mo *K*α radiation, λ = 0.71073 Å
*a* = 7.949 (4) Å	Cell parameters from 3566 reflections
*b* = 10.134 (5) Å	θ = 2.8–28.7°
*c* = 16.444 (8) Å	µ = 0.10 mm^−^^1^
α = 105.43 (5)°	*T* = 293 K
β = 95.37 (4)°	Plate, violet
γ = 102.17 (4)°	0.13 × 0.09 × 0.03 mm
*V* = 1232.4 (11) Å^3^	

### Data collection

**Table:**

Agilent Xcalibur κ-axis diffractometer with a Ruby CCD detector	5284 independent reflections
Radiation source: fine-focus sealed tube	3437 reflections with *I* > 2σ(*I*)
Graphite monochromator	*R*_int_ = 0.018
ω scans	θ_max_ = 27.0°, θ_min_ = 2.8°
Absorption correction: multi-scan (*CrysAlis PRO*; Agilent, 2011)	*h* = −10→10
*T*_min_ = 0.981, *T*_max_ = 1.000	*k* = −12→12
10625 measured reflections	*l* = −20→20

### Refinement

**Table:**

Refinement on *F*^2^	Primary atom site location: structure-invariant direct methods
Least-squares matrix: full	Secondary atom site location: difference Fourier map
*R*[*F*^2^ > 2σ(*F*^2^)] = 0.086	Hydrogen site location: inferred from neighbouring sites
*wR*(*F*^2^) = 0.275	H-atom parameters constrained
*S* = 1.10	*w* = 1/[σ^2^(*F*_o_^2^) + (0.1381*P*)^2^ + 0.6248*P*] where *P* = (*F*_o_^2^ + 2*F*_c_^2^)/3
5284 reflections	(Δ/σ)_max_ = 0.002
322 parameters	Δρ_max_ = 0.60 e Å^−^^3^
9 restraints	Δρ_min_ = −0.49 e Å^−^^3^

### Special details

**Table:**

Geometry. All e.s.d.'s (except the e.s.d. in the dihedral angle between two l.s. planes) are estimated using the full covariance matrix. The cell e.s.d.'s are taken into account individually in the estimation of e.s.d.'s in distances, angles and torsion angles; correlations between e.s.d.'s in cell parameters are only used when they are defined by crystal symmetry. An approximate (isotropic) treatment of cell e.s.d.'s is used for estimating e.s.d.'s involving l.s. planes.
Refinement. Refinement of *F*^2^ against ALL reflections. The weighted *R*-factor *wR* and goodness of fit *S* are based on *F*^2^, conventional *R*-factors *R* are based on *F*, with *F* set to zero for negative *F*^2^. The threshold expression of *F*^2^ > σ(*F*^2^) is used only for calculating *R*-factors(gt) *etc*. and is not relevant to the choice of reflections for refinement. *R*-factors based on *F*^2^ are statistically about twice as large as those based on *F*, and *R*- factors based on ALL data will be even larger.

### Fractional atomic coordinates and isotropic or equivalent isotropic displacement parameters (Å^2^)

**Table:**

	*x*	*y*	*z*	*U*_iso_*/*U*_eq_	Occ. (<1)
O1	1.0682 (3)	1.0348 (3)	−0.1297 (2)	0.0828 (9)	
O2	1.1718 (4)	0.8612 (4)	−0.1166 (3)	0.1000 (11)	
N1	0.2102 (3)	0.6614 (2)	0.06866 (14)	0.0365 (5)	
H1N	0.1178	0.6023	0.0377	0.044*	0.50
N2	1.0538 (4)	0.9210 (4)	−0.11673 (19)	0.0624 (8)	
C1	0.2304 (4)	0.7297 (3)	0.15411 (17)	0.0389 (6)	
C2	0.4064 (4)	0.8181 (3)	0.18213 (19)	0.0470 (7)	
H2	0.4545	0.8742	0.2375	0.056*	
C3	0.4871 (4)	0.8038 (3)	0.11299 (18)	0.0447 (7)	
H3	0.6008	0.8495	0.1116	0.054*	
C4	0.3650 (3)	0.7047 (3)	0.04161 (17)	0.0365 (6)	
C5	0.0641 (4)	0.3740 (3)	−0.18184 (17)	0.0419 (7)	
C6	0.5738 (3)	0.7244 (3)	−0.06002 (17)	0.0374 (6)	
C7	0.6008 (4)	0.8585 (3)	−0.0698 (2)	0.0485 (7)	
H7	0.5121	0.9057	−0.0639	0.058*	
C8	0.7578 (4)	0.9233 (3)	−0.0882 (2)	0.0515 (8)	
H8	0.7754	1.0134	−0.0946	0.062*	
C9	0.8864 (4)	0.8526 (3)	−0.09695 (19)	0.0464 (7)	
C10	0.8646 (5)	0.7199 (4)	−0.0880 (3)	0.0639 (10)	
H10	0.9540	0.6736	−0.0939	0.077*	
C11	0.7068 (5)	0.6564 (4)	−0.0701 (3)	0.0628 (10)	
H11	0.6898	0.5656	−0.0648	0.075*	
O3	−0.2626 (6)	−0.1809 (4)	−0.5712 (2)	0.1246 (15)	
O4	−0.3322 (7)	−0.0114 (5)	−0.6082 (2)	0.1343 (16)	
N3	0.1296 (3)	0.4707 (2)	−0.10375 (14)	0.0398 (6)	
H3N	0.0770	0.4832	−0.0599	0.048*	0.50
N4A	−0.2790 (5)	−0.0618 (4)	−0.5550 (2)	0.0833 (11)	
C12	0.4023 (4)	0.6543 (3)	−0.04098 (17)	0.0373 (6)	
C13	0.2928 (4)	0.5441 (3)	−0.10724 (17)	0.0397 (6)	
C14	0.3327 (4)	0.4883 (3)	−0.1909 (2)	0.0517 (8)	
H14	0.4360	0.5183	−0.2104	0.062*	
C15	0.1940 (4)	0.3851 (3)	−0.2362 (2)	0.0535 (8)	
H15	0.1843	0.3306	−0.2925	0.064*	
C16	−0.1031 (4)	0.2835 (3)	−0.20674 (17)	0.0398 (6)	
C17	−0.1495 (4)	0.1928 (3)	−0.29832 (18)	0.0439 (7)	
C18	−0.1154 (6)	0.0617 (4)	−0.3218 (2)	0.0667 (10)	
H18	−0.0624	0.0291	−0.2808	0.080*	
C19	−0.1593 (6)	−0.0226 (4)	−0.4062 (2)	0.0733 (11)	
H19	−0.1383	−0.1122	−0.4218	0.088*	
C20	−0.2330 (5)	0.0277 (4)	−0.4652 (2)	0.0590 (9)	
C21	−0.2655 (6)	0.1580 (5)	−0.4446 (2)	0.0796 (12)	
H21	−0.3136	0.1917	−0.4863	0.095*	
C22	−0.2252 (6)	0.2392 (4)	−0.3599 (2)	0.0731 (11)	
H22	−0.2502	0.3273	−0.3445	0.088*	
O5	0.9853 (9)	0.2373 (9)	0.3161 (7)	0.233 (4)	
O6	1.0580 (9)	0.4623 (10)	0.3993 (6)	0.233 (4)	
N5	0.9530 (10)	0.3478 (10)	0.3562 (6)	0.160 (3)	
C23	0.7681 (10)	0.3458 (8)	0.3540 (4)	0.1216 (9)	
C24	0.7255 (9)	0.4653 (7)	0.4008 (4)	0.1216 (9)	
H24	0.8110	0.5438	0.4341	0.146*	
C25	0.5530 (9)	0.4635 (8)	0.3964 (4)	0.1216 (9)	
H25	0.5192	0.5424	0.4267	0.146*	
C26	0.4316 (9)	0.3488 (7)	0.3485 (4)	0.1216 (9)	
H26	0.3142	0.3487	0.3468	0.146*	
C27	0.4768 (9)	0.2347 (8)	0.3033 (4)	0.1216 (9)	
H27	0.3897	0.1569	0.2705	0.146*	
C28	0.6424 (9)	0.2289 (8)	0.3038 (4)	0.1216 (9)	
H28	0.6724	0.1493	0.2716	0.146*	

### Atomic displacement parameters (Å^2^)

**Table:**

	*U*^11^	*U*^22^	*U*^33^	*U*^12^	*U*^13^	*U*^23^
O1	0.0524 (16)	0.099 (2)	0.104 (2)	−0.0040 (15)	0.0200 (15)	0.0572 (19)
O2	0.0414 (15)	0.127 (3)	0.145 (3)	0.0250 (17)	0.0367 (17)	0.050 (2)
N1	0.0361 (12)	0.0349 (11)	0.0321 (11)	0.0014 (9)	0.0077 (9)	0.0039 (9)
N2	0.0354 (15)	0.089 (2)	0.0634 (19)	0.0058 (15)	0.0115 (13)	0.0285 (17)
C1	0.0405 (15)	0.0338 (13)	0.0354 (14)	0.0018 (11)	0.0061 (11)	0.0042 (11)
C2	0.0429 (17)	0.0450 (16)	0.0393 (16)	−0.0023 (13)	0.0003 (12)	0.0017 (12)
C3	0.0404 (16)	0.0429 (15)	0.0416 (16)	−0.0006 (12)	0.0056 (12)	0.0057 (12)
C4	0.0353 (14)	0.0331 (13)	0.0382 (14)	0.0038 (11)	0.0064 (11)	0.0093 (11)
C5	0.0469 (17)	0.0419 (15)	0.0324 (14)	0.0054 (13)	0.0103 (12)	0.0061 (11)
C6	0.0349 (14)	0.0410 (14)	0.0348 (14)	0.0072 (11)	0.0076 (11)	0.0093 (11)
C7	0.0362 (16)	0.0450 (16)	0.068 (2)	0.0103 (13)	0.0155 (14)	0.0203 (15)
C8	0.0422 (17)	0.0471 (17)	0.071 (2)	0.0068 (14)	0.0139 (15)	0.0280 (16)
C9	0.0315 (15)	0.0611 (19)	0.0457 (17)	0.0052 (14)	0.0086 (12)	0.0180 (14)
C10	0.0454 (19)	0.070 (2)	0.091 (3)	0.0261 (17)	0.0242 (18)	0.035 (2)
C11	0.053 (2)	0.0543 (19)	0.097 (3)	0.0200 (16)	0.0247 (19)	0.0391 (19)
O3	0.189 (4)	0.089 (2)	0.065 (2)	0.023 (3)	0.023 (2)	−0.0227 (18)
O4	0.194 (5)	0.139 (3)	0.0400 (17)	0.018 (3)	−0.016 (2)	0.0073 (19)
N3	0.0421 (13)	0.0399 (12)	0.0305 (12)	0.0017 (10)	0.0091 (9)	0.0044 (9)
N4A	0.089 (3)	0.088 (3)	0.0426 (19)	−0.008 (2)	0.0128 (17)	−0.0086 (18)
C12	0.0371 (15)	0.0358 (13)	0.0384 (14)	0.0052 (11)	0.0097 (11)	0.0117 (11)
C13	0.0418 (16)	0.0400 (14)	0.0354 (14)	0.0037 (12)	0.0112 (11)	0.0110 (12)
C14	0.0487 (18)	0.0564 (18)	0.0428 (17)	0.0009 (15)	0.0197 (14)	0.0077 (14)
C15	0.059 (2)	0.0539 (18)	0.0362 (15)	0.0014 (15)	0.0201 (14)	−0.0002 (13)
C16	0.0465 (16)	0.0376 (14)	0.0298 (13)	0.0058 (12)	0.0077 (11)	0.0035 (11)
C17	0.0445 (16)	0.0445 (15)	0.0337 (14)	0.0004 (13)	0.0088 (12)	0.0040 (12)
C18	0.102 (3)	0.054 (2)	0.0382 (17)	0.025 (2)	0.0027 (18)	0.0029 (15)
C19	0.102 (3)	0.056 (2)	0.050 (2)	0.019 (2)	0.013 (2)	−0.0049 (17)
C20	0.061 (2)	0.064 (2)	0.0318 (16)	−0.0046 (17)	0.0078 (14)	−0.0035 (14)
C21	0.107 (3)	0.080 (3)	0.042 (2)	0.020 (2)	−0.007 (2)	0.0106 (18)
C22	0.106 (3)	0.064 (2)	0.0422 (19)	0.030 (2)	0.0006 (19)	0.0017 (16)
O5	0.164 (6)	0.203 (7)	0.357 (12)	0.088 (6)	0.022 (6)	0.098 (8)
O6	0.134 (5)	0.259 (9)	0.256 (8)	−0.048 (6)	−0.039 (5)	0.089 (7)
N5	0.129 (6)	0.158 (6)	0.217 (8)	0.055 (5)	0.040 (5)	0.078 (6)
C23	0.128 (2)	0.138 (2)	0.106 (2)	0.0241 (19)	0.0334 (17)	0.0501 (17)
C24	0.128 (2)	0.138 (2)	0.106 (2)	0.0241 (19)	0.0334 (17)	0.0501 (17)
C25	0.128 (2)	0.138 (2)	0.106 (2)	0.0241 (19)	0.0334 (17)	0.0501 (17)
C26	0.128 (2)	0.138 (2)	0.106 (2)	0.0241 (19)	0.0334 (17)	0.0501 (17)
C27	0.128 (2)	0.138 (2)	0.106 (2)	0.0241 (19)	0.0334 (17)	0.0501 (17)
C28	0.128 (2)	0.138 (2)	0.106 (2)	0.0241 (19)	0.0334 (17)	0.0501 (17)

### Geometric parameters (Å, º)

**Table:**

O1—N2	1.212 (4)	C12—C13	1.399 (4)
O2—N2	1.220 (4)	C13—C14	1.435 (4)
N1—C1	1.369 (3)	C14—C15	1.345 (5)
N1—C4	1.372 (3)	C14—H14	0.9300
N1—H1N	0.8600	C15—H15	0.9300
N2—C9	1.472 (4)	C16—C1^i^	1.399 (4)
C1—C16^i^	1.399 (4)	C16—C17	1.505 (4)
C1—C2	1.451 (4)	C17—C22	1.366 (5)
C2—C3	1.347 (4)	C17—C18	1.374 (5)
C2—H2	0.9300	C18—C19	1.390 (5)
C3—C4	1.441 (4)	C18—H18	0.9300
C3—H3	0.9300	C19—C20	1.353 (6)
C4—C12	1.401 (4)	C19—H19	0.9300
C5—N3	1.367 (4)	C20—C21	1.359 (6)
C5—C16	1.400 (4)	C21—C22	1.384 (5)
C5—C15	1.433 (4)	C21—H21	0.9300
C6—C11	1.379 (4)	C22—H22	0.9300
C6—C7	1.386 (4)	O5—N5	1.230 (9)
C6—C12	1.500 (4)	O6—N5	1.260 (9)
C7—C8	1.382 (4)	N5—C23	1.462 (10)
C7—H7	0.9300	C23—C24	1.378 (8)
C8—C9	1.363 (4)	C23—C28	1.382 (8)
C8—H8	0.9300	C24—C25	1.363 (8)
C9—C10	1.368 (5)	C24—H24	0.9300
C10—C11	1.379 (5)	C25—C26	1.342 (8)
C10—H10	0.9300	C25—H25	0.9300
C11—H11	0.9300	C26—C27	1.337 (8)
O3—N4A	1.203 (5)	C26—H26	0.9300
O4—N4A	1.210 (5)	C27—C28	1.329 (8)
N3—C13	1.367 (4)	C27—H27	0.9300
N3—H3N	0.8600	C28—H28	0.9300
N4A—C20	1.477 (4)		
			
C1—N1—C4	107.2 (2)	C12—C13—C14	125.8 (3)
C1—N1—H1N	126.4	C15—C14—C13	107.8 (3)
C4—N1—H1N	126.4	C15—C14—H14	126.1
O1—N2—O2	123.8 (3)	C13—C14—H14	126.1
O1—N2—C9	118.5 (3)	C14—C15—C5	107.6 (3)
O2—N2—C9	117.7 (3)	C14—C15—H15	126.2
N1—C1—C16^i^	126.0 (3)	C5—C15—H15	126.2
N1—C1—C2	109.0 (2)	C1^i^—C16—C5	125.8 (3)
C16^i^—C1—C2	125.0 (3)	C1^i^—C16—C17	117.4 (3)
C3—C2—C1	107.1 (3)	C5—C16—C17	116.9 (2)
C3—C2—H2	126.4	C22—C17—C18	118.6 (3)
C1—C2—H2	126.4	C22—C17—C16	120.9 (3)
C2—C3—C4	107.4 (3)	C18—C17—C16	120.5 (3)
C2—C3—H3	126.3	C17—C18—C19	120.6 (3)
C4—C3—H3	126.3	C17—C18—H18	119.7
N1—C4—C12	125.5 (2)	C19—C18—H18	119.7
N1—C4—C3	109.2 (2)	C20—C19—C18	118.9 (3)
C12—C4—C3	125.2 (3)	C20—C19—H19	120.6
N3—C5—C16	126.6 (3)	C18—C19—H19	120.6
N3—C5—C15	108.2 (3)	C19—C20—C21	122.1 (3)
C16—C5—C15	125.2 (3)	C19—C20—N4A	118.8 (4)
C11—C6—C7	118.3 (3)	C21—C20—N4A	119.1 (4)
C11—C6—C12	121.2 (3)	C20—C21—C22	118.3 (4)
C7—C6—C12	120.5 (2)	C20—C21—H21	120.9
C8—C7—C6	120.9 (3)	C22—C21—H21	120.9
C8—C7—H7	119.5	C17—C22—C21	121.5 (4)
C6—C7—H7	119.5	C17—C22—H22	119.2
C9—C8—C7	118.9 (3)	C21—C22—H22	119.2
C9—C8—H8	120.6	O5—N5—O6	128.7 (9)
C7—C8—H8	120.6	O5—N5—C23	115.7 (9)
C8—C9—C10	122.0 (3)	O6—N5—C23	115.6 (8)
C8—C9—N2	119.1 (3)	C24—C23—C28	121.9 (7)
C10—C9—N2	118.9 (3)	C24—C23—N5	117.8 (7)
C9—C10—C11	118.5 (3)	C28—C23—N5	120.3 (7)
C9—C10—H10	120.8	C25—C24—C23	117.2 (7)
C11—C10—H10	120.8	C25—C24—H24	121.4
C6—C11—C10	121.5 (3)	C23—C24—H24	121.4
C6—C11—H11	119.3	C26—C25—C24	120.6 (7)
C10—C11—H11	119.3	C26—C25—H25	119.7
C13—N3—C5	108.3 (2)	C24—C25—H25	119.7
C13—N3—H3N	125.8	C27—C26—C25	120.9 (7)
C5—N3—H3N	125.8	C27—C26—H26	119.5
O3—N4A—O4	123.5 (4)	C25—C26—H26	119.5
O3—N4A—C20	118.4 (4)	C28—C27—C26	122.0 (8)
O4—N4A—C20	118.2 (4)	C28—C27—H27	119.0
C13—C12—C4	125.4 (3)	C26—C27—H27	119.0
C13—C12—C6	117.2 (2)	C27—C28—C23	117.4 (7)
C4—C12—C6	117.3 (2)	C27—C28—H28	121.3
N3—C13—C12	126.2 (2)	C23—C28—H28	121.3
N3—C13—C14	108.1 (2)		
			
C4—N1—C1—C16^i^	179.5 (3)	N3—C13—C14—C15	0.8 (4)
C4—N1—C1—C2	−0.9 (3)	C12—C13—C14—C15	−179.2 (3)
N1—C1—C2—C3	1.3 (3)	C13—C14—C15—C5	0.1 (4)
C16^i^—C1—C2—C3	−179.1 (3)	N3—C5—C15—C14	−1.0 (4)
C1—C2—C3—C4	−1.2 (3)	C16—C5—C15—C14	176.5 (3)
C1—N1—C4—C12	176.6 (3)	N3—C5—C16—C1^i^	−3.9 (5)
C1—N1—C4—C3	0.2 (3)	C15—C5—C16—C1^i^	179.1 (3)
C2—C3—C4—N1	0.7 (3)	N3—C5—C16—C17	176.0 (3)
C2—C3—C4—C12	−175.8 (3)	C15—C5—C16—C17	−1.0 (5)
C11—C6—C7—C8	−0.8 (5)	C1^i^—C16—C17—C22	89.5 (4)
C12—C6—C7—C8	−179.3 (3)	C5—C16—C17—C22	−90.4 (4)
C6—C7—C8—C9	0.2 (5)	C1^i^—C16—C17—C18	−90.9 (4)
C7—C8—C9—C10	0.0 (5)	C5—C16—C17—C18	89.2 (4)
C7—C8—C9—N2	179.9 (3)	C22—C17—C18—C19	−0.9 (6)
O1—N2—C9—C8	−4.4 (5)	C16—C17—C18—C19	179.5 (3)
O2—N2—C9—C8	173.5 (3)	C17—C18—C19—C20	1.3 (6)
O1—N2—C9—C10	175.5 (3)	C18—C19—C20—C21	0.0 (6)
O2—N2—C9—C10	−6.6 (5)	C18—C19—C20—N4A	179.8 (4)
C8—C9—C10—C11	0.4 (6)	O3—N4A—C20—C19	6.1 (6)
N2—C9—C10—C11	−179.5 (3)	O4—N4A—C20—C19	−174.3 (4)
C7—C6—C11—C10	1.2 (5)	O3—N4A—C20—C21	−174.1 (4)
C12—C6—C11—C10	179.7 (3)	O4—N4A—C20—C21	5.5 (6)
C9—C10—C11—C6	−1.0 (6)	C19—C20—C21—C22	−1.6 (7)
C16—C5—N3—C13	−176.0 (3)	N4A—C20—C21—C22	178.6 (4)
C15—C5—N3—C13	1.4 (3)	C18—C17—C22—C21	−0.8 (6)
N1—C4—C12—C13	−5.3 (5)	C16—C17—C22—C21	178.8 (4)
C3—C4—C12—C13	170.6 (3)	C20—C21—C22—C17	2.1 (7)
N1—C4—C12—C6	174.7 (2)	O5—N5—C23—C24	177.4 (8)
C3—C4—C12—C6	−9.5 (4)	O6—N5—C23—C24	−2.3 (10)
C11—C6—C12—C13	−71.9 (4)	O5—N5—C23—C28	−4.8 (11)
C7—C6—C12—C13	106.6 (3)	O6—N5—C23—C28	175.5 (7)
C11—C6—C12—C4	108.1 (3)	C28—C23—C24—C25	0.6 (8)
C7—C6—C12—C4	−73.4 (4)	N5—C23—C24—C25	178.3 (6)
C5—N3—C13—C12	178.6 (3)	C23—C24—C25—C26	0.5 (8)
C5—N3—C13—C14	−1.4 (3)	C24—C25—C26—C27	−0.9 (9)
C4—C12—C13—N3	2.5 (5)	C25—C26—C27—C28	0.3 (10)
C6—C12—C13—N3	−177.5 (2)	C26—C27—C28—C23	0.7 (10)
C4—C12—C13—C14	−177.5 (3)	C24—C23—C28—C27	−1.1 (9)
C6—C12—C13—C14	2.5 (4)	N5—C23—C28—C27	−178.8 (6)

Symmetry code: (i) −*x*, −*y*+1, −*z*.

### Hydrogen-bond geometry (Å, º)

**Table:**

*D*—H···*A*	*D*—H	H···*A*	*D*···*A*	*D*—H···*A*
C3—H3···O1^ii^	0.93	2.60	3.519 (4)	171
C11—H11···N1^iii^	0.93	2.57	3.426 (4)	152
C15—H15···O3^iv^	0.93	2.56	3.453 (5)	161
C18—H18···O1^v^	0.93	2.58	3.455 (5)	157
C26—H26···O5^vi^	0.93	2.56	3.432 (10)	156

Symmetry codes: (ii) −*x*+2, −*y*+2, −*z*; (iii) −*x*+1, −*y*+1, −*z*; (iv) −*x*, −*y*, −*z*−1; (v) *x*−1, *y*−1, *z*; (vi) *x*−1, *y*, *z*.
