# Supplementary figures and images for: Crystal structure of meso-tetra­kis­(4-nitro­phen­yl)porphyrin nitro­benzene disolvate
Source: Acta Crystallogr Sect E Struct Rep Online. 2014 Oct 4;70(Pt 11):o1147–8. doi: 10.1107/S1600536814021503 (PMC4257332; doi:10.1107/S1600536814021503)

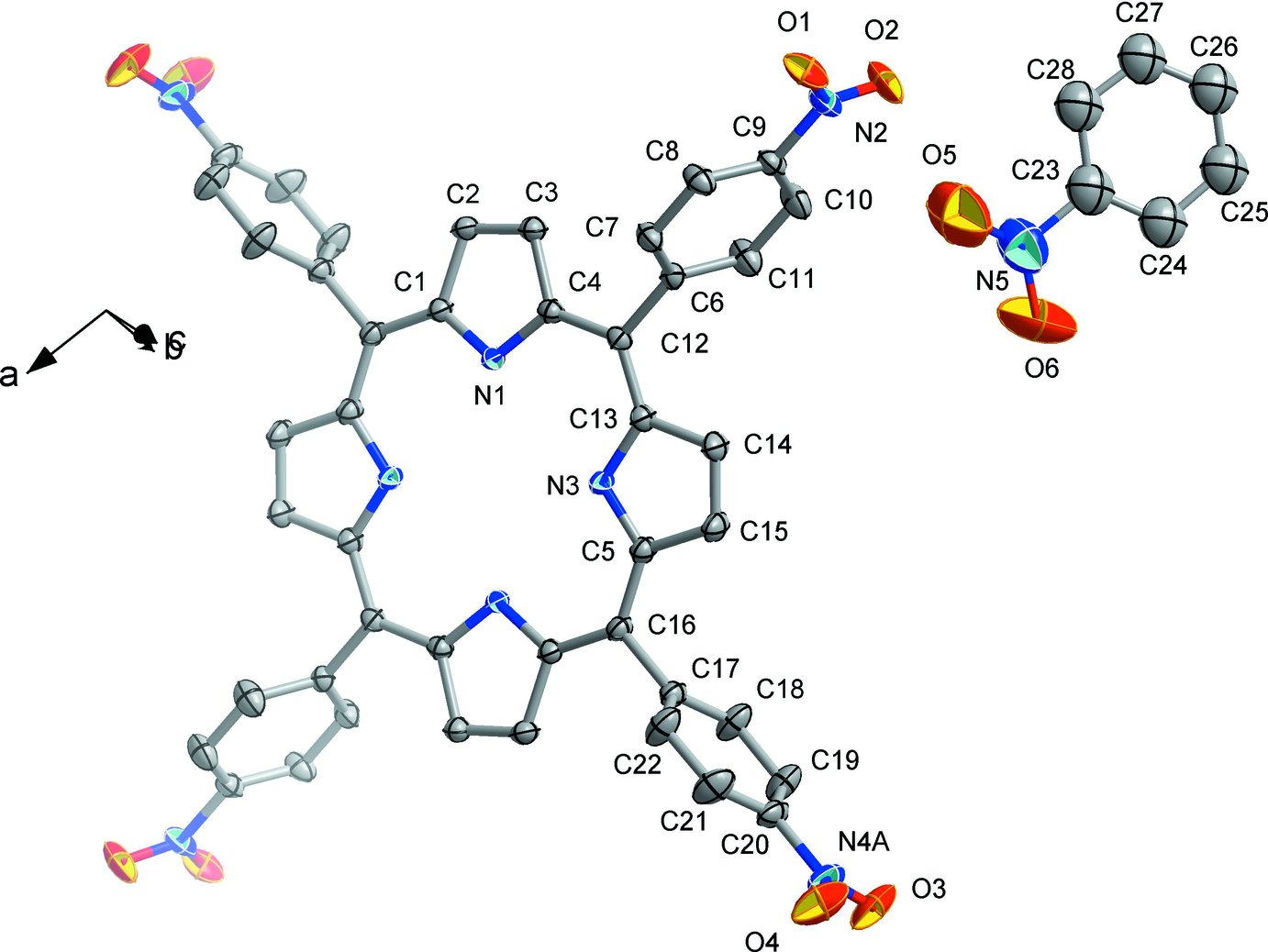

Supplement: Supplementary file 4 [file e-70-o1147-fig1.tif]

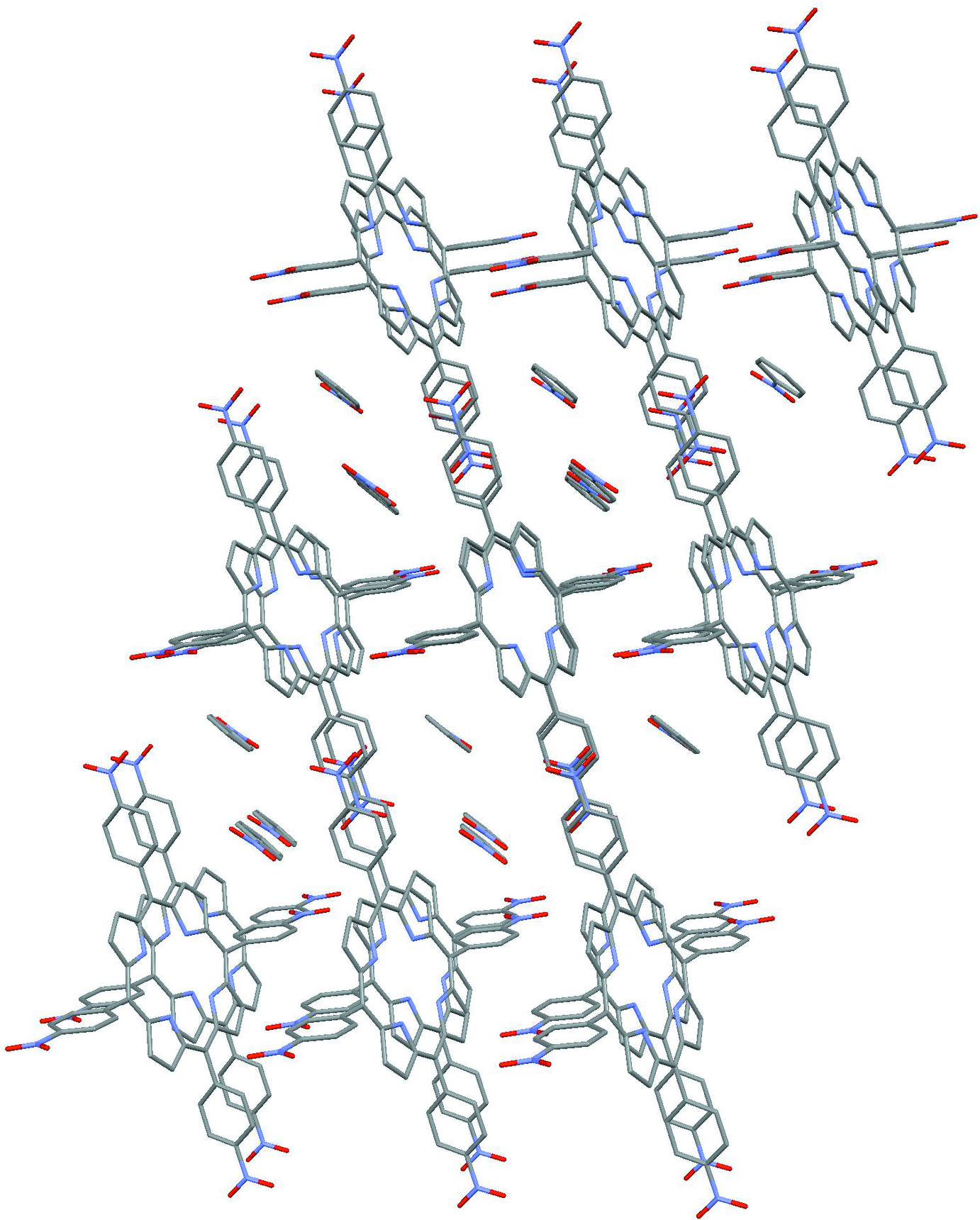

Supplement: Supplementary file 5 [file e-70-o1147-fig2.tif]
